# Potential of *GJA8* gene variants in predicting age-related cataract: A comparison of supervised machine learning methods

**DOI:** 10.1371/journal.pone.0286243

**Published:** 2023-08-31

**Authors:** Saba Zafar, Haris Khurram, Muhammad Kamran, Madeeha Fatima, Aqsa Parvaiz, Rehan Sadiq Shaikh

**Affiliations:** 1 Department of Biochemistry and Biotechnology, The Women University, Multan, Pakistan; 2 Institute of Molecular Biology and Biotechnology, Bahauddin Zakariya University, Multan, Pakistan; 3 Department of Sciences and Humanities, National University of Computer and Emerging Sciences, Chiniot-Faisalabad Campus, Chiniot, Pakistan; 4 Department of Medical Laboratory Technology, Islamabad Medical & Dental college, Islamabad, Pakistan; 5 Department of Zoology, The Women University, Multan, Pakistan; 6 Center for Applied Molecular Biology, University of the Punjab, Lahore, Pakistan; University of North Carolina at Chapel Hill, UNITED STATES

## Abstract

Cataracts are the problems associated with the crystallins proteins of the eye lens. Any perturbation in the conformity of these proteins results in a cataract. Age-related cataract is the most common type among all cataracts as it accounts for almost 80% of cases of senile blindness worldwide. This research study was performed to predict the role of single nucleotide polymorphisms (SNPs) of the *GJA8* gene with age-related cataracts in 718 subjects (400 age-related cataract patients and 318 healthy individuals). A comparison of supervised machine learning classification algorithm including logistic regression (LR), random forest (RF) and Artificial Neural Network (ANN) were presented to predict the age-related cataracts. The results indicated that LR is the best for predicting age-related cataracts. This successfully developed model after accounting different genetic and demographic factors to predict cataracts will help in effective disease management and decision-making medical practitioner and experts.

## Introduction

A cataract is a cloudy patch that appears in the eye lens and leads to reduced vision clarity and eventually blindness. This condition can result in both reversible and irreversible visual impairments [1]. Globally, cataracts are the second most common visual impairment after lens refractive errors. Crystallins are the primary proteins involved in the formation of the lens and its outer layer. Any structural or functional modification in crystallins, their accumulation, precipitation, and breakdown lead to cataract formation. Cataracts can be characterized into several types depending upon the causative agent and clinical condition. For instance, there are age-related cataracts, childhood cataracts, secondary cataracts, and morphologically classified cataracts. Age-related cataracts are the most common type of cataracts which develop in individuals above 50 years of age. Childhood cataracts include congenital cataracts (at birth) and juvenile cataracts (during childhood). Secondary cataracts occur due to eye infection or glaucoma or due to side effects of some drugs such as corticosteroids. Morphologically, cataracts can be further classified into seven more categories. These categories include nuclear cataracts, supra nuclear cataracts, cortical cataracts, sutural cataracts, capsular cataracts, subcapsular cataracts, and lamellar cataracts [2–4]. Table 1 describes the different categories of cataract.

**Table 1:** Classifications of different types of cataracts.

Morphological classification	Classification according to maturity	Etiological classification
Capsular cataract	Immature cataract	Congenital cataract
Subcapsular cataract	Mature cataract	Developmental cataract
Cortical cataract	Hyper mature cataract	Acquired cataract
Nuclear cataract	Intumescent cataract	
Supra nuclear cataract		
Lamellar cataract		
Sutural cataract		
Polar Cataract		
Membranous		
Punctate Cataract		
Mixed Cataract		
Total Cataract		

However, age-related cataract is the most common type. About 80% of cataract cases are age-related, even though congenital cataracts are very harmful to eyesight. Eye lens with increasing age gradually becomes opaque, which blocks the transmission of light from the eyeball to the retina. This condition is known as an age-related cataract. Multiple environmental and genetic risk factors are associated with age-related cataract progression. Systematic studies have indicated that age-related cataract is a heterogeneous disorder; thus, it is caused by interactions of several genetic and environmental factors such as age, gender, ethnicity, diet, smoking, radiation, and diabetes [5–9]. However, the genetic risk factor, which accounts for half of the risk, is gaining more attention and recognition as being the most important determinant of age-related cataract development [3,4]. Twin studies have revealed a heritability of 48% for nuclear and 50% for cortical subtypes of age-related cataracts [10,11]. Most of the research work has been related to inherited congenital cataracts; despite their best efforts, prevalent genetic variants associated with age-related cataracts have yet to be discovered. Case-control correlational studies have investigated the genetic predisposition to age-related cataracts. Through the most commonly used technique i.e., the candidate gene approach to identify genetic factors, research studies have revealed that various polymorphisms in coding and non-coding region of genes are an underlying cause of hereditary cataract. These genetic variations are also linked to age-related cataract. Polymorphisms in *EPHA2*, *GJA3*, *GJA8*, *MIP*, *HSF4*, *LIM2*, and *CRYAA*, *CRYAB and CRYBB1* genes found to be associated with development and progression of age-related cataract along with congenital cataract [12–16]. There are some other genes associated with age related cataract including *GSTM1*, *GSTT1*, *ERCC2*, *MTHFR*, *LCT*, *FTO*, *APOE4*, *KLC1* and *ARCC1* [17–20]. However, variation in these genes is not associated with congenital cataracts. Association of only *EPHA2* gene with age-related cataracts has been studied in different populations including Chinese and Indians [21–23]. The association of the *GJA8* gene with age-related cataracts has been studied only in the Chinese population [24,25].

Connexin50 is a gap junction protein found in the eye lens and is encoded by the gap junction alpha8 (*GJA8*) gene (located at the lq21.1 chromosome). It is required to transport tiny molecules (like second messengers, metabolites, ions, and water) from the surface of the outer retina towards the central lens nucleus [26]. Several *GJA8* mutations have been linked to hereditary cataracts. Studies have demonstrated that till now above 20 variants of the *GJA8* gene have been found in congenital cataract pedigrees from all around the world, the majority of which are inherited via autosomal dominance. However, limited studies reported an association of *GJA8* gene variants with age-related cataracts. Targeted mutation of the *GJA8* gene in rats resulted in the development of microphthalmia and zonular pulverulent cataracts [27]. The Beaver Dam Eye Study has discovered numerous SNPs associated with age-related cortical cataracts via genome sequencing analysis. Although the loci of these SNPs did not match the mapping locations of *GJA8*, the research demonstrates that the weaker loci might serve as regulators and can be important for the development of age-related cataracts [28]. In previous research, a unique *GJA8* genetic variation linked to autosomal dominant cataracts was discovered in a five-generation pedigree [29]. So, extensive research has been carried out to investigate the *GJA8* gene and its polymorphisms in congenital cataracts, more research should be done to identify the role of the *GJA8* gene in age-related cataracts. Identifying SNPs as a genetic risk factor for age-related cataracts is important for effective diagnosis and treatment of the disease.

As age-related cataract has emerged as a severe concern in ophthalmology in the past few years because of their socioeconomic effect as well as the increasing incidence and severity of this disease. Thus, a more accurate and reliable diagnostic approach is required to detect the incidence of age-related cataract development at an earlier stage. Traditional regression approaches are often employed to build predictive models that help practitioners diagnose age-related cataracts. These approaches necessitate selecting variables based on prior data distribution assumptions throughout the development process, which inevitably results in the loss of information [30].

Recently, machine learning and dimensionality reduction approaches have been discovered to be quite efficient for diagnostic purposes and identifying high-risk factors contributing to disease progression. Machine learning approaches stand out as a useful and reliable tool for predictive medicine [31]. However, only a few machine learning models are present for cataract development prediction.

Thus, this study aimed to predict the role of SNPs rs9437983 and rs1495960 of the *GJA8* gene in onset age-related cataracts. Furthermore, three machine learning tools (logistic regression (LR), random forest (RF), and artificial neural network (ANN)) integrating SNPs and demographic factors were utilized to predict the earlier detection of age-related cataracts, which would be important for minimizing cataract incidence and improving treatment rates. This will also ultimately help to understand the underlying molecular mechanism of cataracts in the Pakistani population. As rs1495960 and rs9437983 located in *GJA8* gene have been considered as causative variants of ARC in Chinese population and are frequently predisposed to ARC [24], so we selected these variants to predict the ARC in combination with other demographic factors.

## Materials and methods

### Study approval and sample collection

Approval of the study was obtained from the Institutional Review Boards (IRB) of the Institute of Molecular Biology and Biotechnology, Bahauddin Zakariya University, Multan, Pakistan. Participants were recruited from the Department of ophthalmology at Nishtar Hospital, Multan which is a hub for the people of southern Punjab and Baluchistan Province A written consent was obtained from all the participants, including 400 cataract patients (as cases) and 318 healthy individuals (as controls). Blood samples (10cc) were collected from both cases and controls in EDTA-containing tubes and preserved at −80°C for further analysis.

### DNA extraction and quantification

The genomic DNA of collected blood samples was extracted via the previously described inorganic method [32]. UV spectrophotometer (Perkin Elmer Lambda 25) was used to quantify the concentration and purity of extracted DNA. 0.8% agarose gel with ethidium bromide (EtBr) was used to evaluate the integrity of purified DNA.

### Primer designing and amplification of *GJA8* gene by tetra arm PCR and DNA sequencing

The genotyping for the SNPs were detected by tetra amplification-refractory mutation system (ARMS) and confirmed by DNA sequencing. PCR Polymerase chain reaction (PCR) was used to amplify target DNA in the *GJA8* gene via tetra amplification-refractory mutation system (ARMS) PCR. This method used four primers to amplify a target DNA fragment with polymorphism. Primers for amplification purposes were designed so that two distinct alleles produce products of different sizes. It helped easily and accurately visualize different-sized bands separately on an agarose gel electrophoresis. The heterozygous genotypes are characterized by the visualization of 3 different-sized PCR products, whereas the homozygous genotypes are comprised of 2 PCR products on gel electrophoresis. Four primers were designed by PRIMER 1 software for genotyping of targeted DNA. Sequences of all four primers of both SNPs of the *GJA8* gene are given in Table 2.

**Table 2:** Tetra ARMS-Primer sequences designed and employed for the amplification of *GJA8* gene variants.

Gene	Primers	Sequence	Product size	Annealing temperature (⁰C)
***GJA8*: A/G** **rs9437983**	Forward inner primer (G allele)	GGGCTATGCTGTAATGGGCTTAGATAG	212	61
	Reverse inner primer (A allele)	TAGGATGTGAGCCCTGGTCTGCT	280	61
	Forward outer primer (5’ - 3’)	GAGCATTTAATGGAGCACAGGAAGG	414	61
	Reverse outer primer (5’ - 3’)	GGTTGTGGAAACCTTCATCTTGAGC	414	61
***GJA8*: T/G** **rs1495960**	Forward inner primer (T allele)	GCTTAGATCTAGAAGACCAGGGCTCATAT	191	59
	Reverse inner primer (G allele)	CTTAGCTAGTGAGGGCAGAGCTAAGC	268	59
	Forward outer primer (5’- 3’)	TAAGGACCATAAAATTCCTCCTAGGAGC	404	59
	Reverse outer primer (5’- 3’)	GTGGAAACCTTCATCTTGAGCTAGAACT	404	59

**Fig 1:**
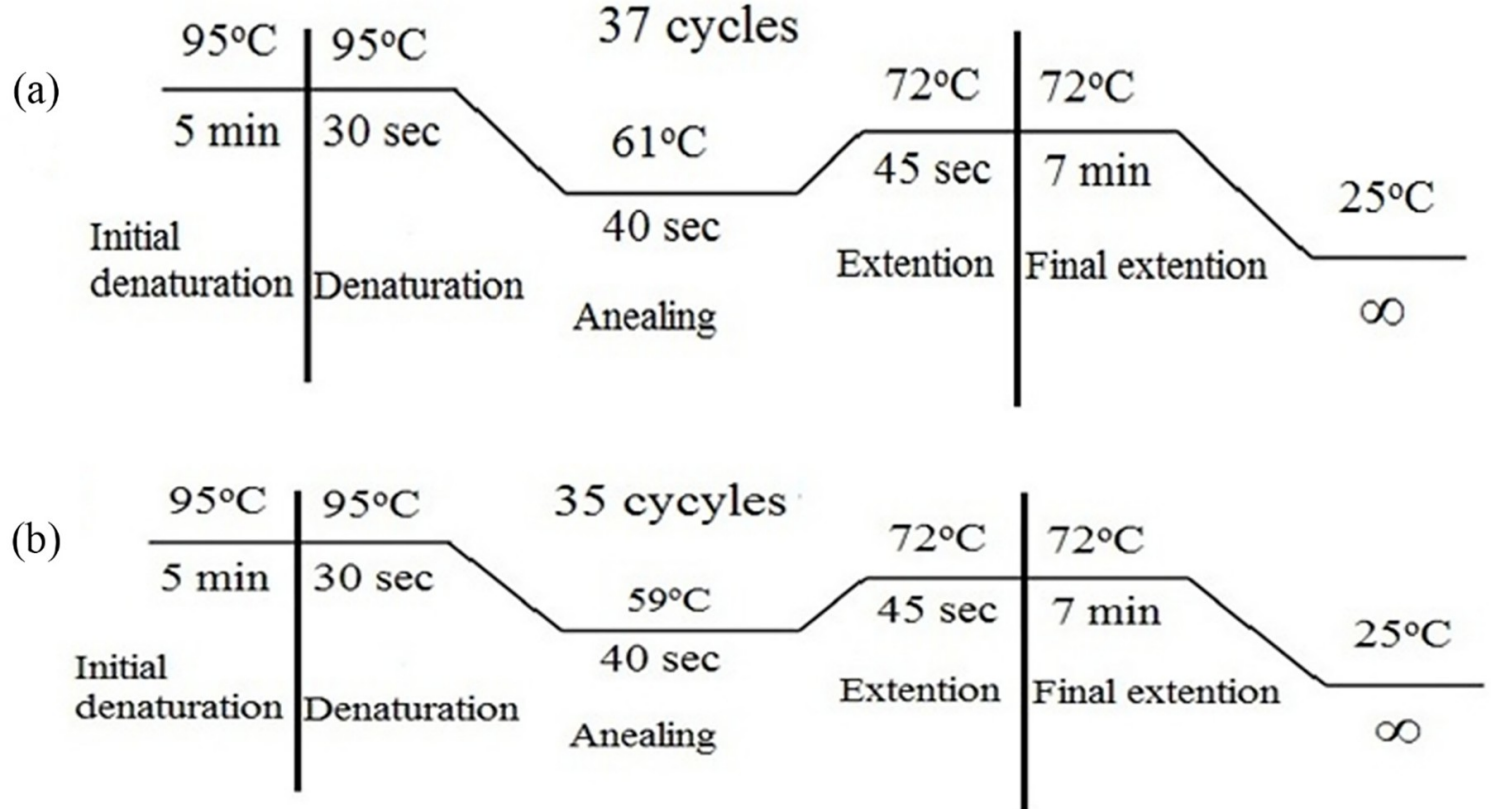
Thermal profile of PCR for a) rs9437983 and b) rs1495960 SNPs.

A reaction mixture of a final volume of 20μl was prepared to perform tetra-ARMS PCR. 20μl of reaction mixture comprised prepared by adding 2ul of 10 X PCR Buffer, 2μl of 2.5mM deoxynucleotide (dNTP) mixture, 0.9μl of 50mM magnesium chloride (MgCl_2_,), 0.3μl Taq polymerase (5U) (Fermentas, UK), 9.3μl PCR water, 0.5μl of each outer and 1μl of each inner primer (10 picomols), 2ul of DNA template from the stock concentration of 25ng and deoxyribonuclease (DNase) free deionized water. Water was used as negative control instead of DNA. DNA amplification was carried out in a DNA thermal cycler (Gene Amp PCR system 2700 Applied Biosystems Inc., UK). The thermo-profile used is given in Fig 1.

### Gel electrophoresis

PCR products were run on 2% agarose gel electrophoresis stained with EtBr to visualize the results. UV trans illuminator was used to visualize bands present on the agarose gel.

### Demographic variables

Previous research studies demonstrated that demographic variables are among the leading risk factors for age-related cataracts. Hence, to better predict the onset of age-related cataracts, different demographic variables were included in this study. The demographic variables included gender, age, body mass index (BMI), diabetes health status, blood pressure, vitamins, tranquilizers, smoking, radiation, and steroid medications were included in a model building along with genetic markers and haplotypes [33–36].

### Data analysis

The data of 718 participants were divided into testing and training parts. 538 (75%) randomly selected observations were used for training the models and the remaining 180 (25%) observations were used as a test case for measuring the accuracy of the model. Two different models were used for the prediction of cataracts. The first model predicts the ARC by the use of demographic factors and *GJA8* SNPs, and the second model predict the ARC by the use of demographic factors and the haplotypes of the *GJA8* gene. The models used in this study are represented below:

Catarect=f(Gender+BMI+H.O.Diabetes+H.O.B.P+Vitamins+Tranquilizers+Smoking+Radiation+Steroids+SNPs) Model 1

Catarect=f(Gender+BMI+H.O.Diabetes+H.O.B.P+Vitamins+Tranquilizers+Smoking+Radiation+Steroids+Haplotype) Model 2

We used different supervised machine learning methods and compared the performance of each model by using different performance evaluation measures including area under the curve (AUC), its 95% confidence interval, sensitivity, and specificity. All the modelling and comparison were performed on R language.

### Supervised machine learning models

#### Logistic regression (LR)

LR is used when a binary outcome is expected, for example, the presence or absence of a cataract. It results in the likelihood or the probability of occurrence or non-occurrence of the diseases. As the outcome is a probability, it is generally bounded between 0 and 1. Both model 1 and model 2 were fitted using the LR method.

#### Artificial neural network (ANN)

Neural network models comprise multiple layers of algorithms where each layer learns and communicates with the other layer regarding handling predictors. Usually, such a model comprises hidden inner layers, where the main computation is carried out. The ANN model was developed with one input and one output layer with two hidden layers in between. Inside a hidden layer, the model algorithm estimates and applies weights to different predictors and directs through an activation algorithm to give the final output. Both models were also fitted using an ANN-based algorithm. Weights are applied to different predictors or pseudo predictors in the hidden layers. These weights help estimate the importance of each predictor in the final output model. For ANN model we used neural net package in R with default setting using five node and three layers. The training of ANN model was done using resilient backpropagation algorithm.

#### Random forest (RF) model

RF models comprise multiple decision trees regarding the explanatory variables during the model learning stage. Afterward, the bagging or bootstrapping step helps decrease the model’s variance without impacting the model’s bias. RF algorithms are pretty suitable for correlated predictors as they help in decorrelating those predictors and better train a model [37]. RF models can be used for classification and regression purposes. Both models were also tested using RF algorithms. For RF model we used random Forest package.

### Model comparison criteria

The model performance is generally explained in terms of how well a model can classify or differentiate the problem under consideration. In order to compare the performance of LR, ANN, and RF models Area Under the Curve (AUC) and Receiver Operating Characteristics (ROC) were used. AUC gives the measure of separability, and ROC gives the probability of an outcome belonging one class to another, i.e., cataract or cataract free in this case. A higher AUC value indicates a better model. To ensure validation of the performance of each model, 10-fold cross validation was done.

## Results

**Fig 2:**
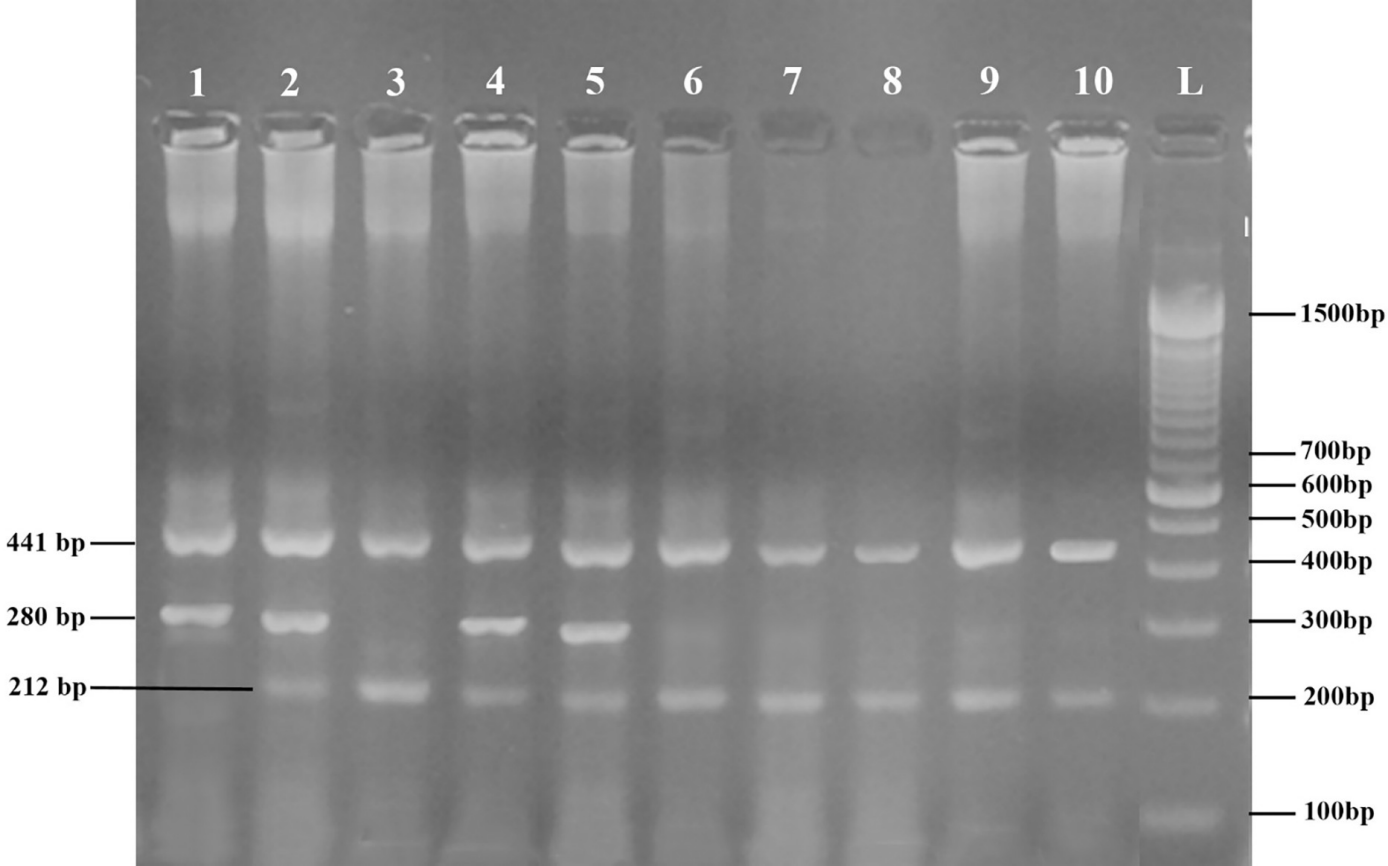
PCR products on ethidium bromide-stained electrophoresis gel. Homozygous genotype AA (lane 1); homozygous GG genotype (lane 3,6,7,8,9,10); heterozygous A/G genotype (lane 2, 4, 5); for GJA8 rs9437983.

**Fig 3:**
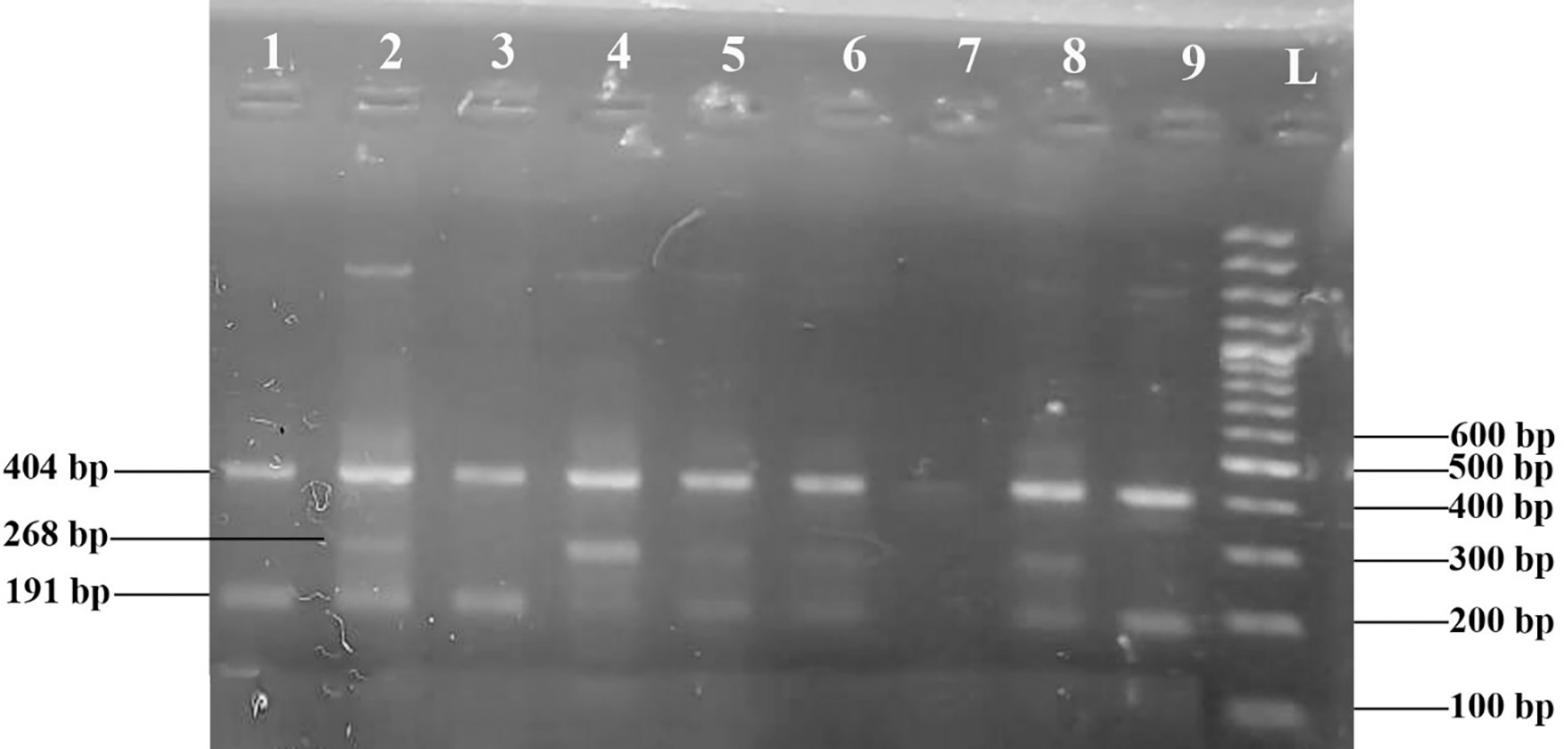
PCR products on ethidium bromide electrophoresis gel. Homozygous TT genotype (lane 1, 3, 9); heterozygous T/G genotype (lane 2,5,6,8,9); homozygous GG (lane 4) genotype for GJA8 rs1495960.

**Fig 4:**
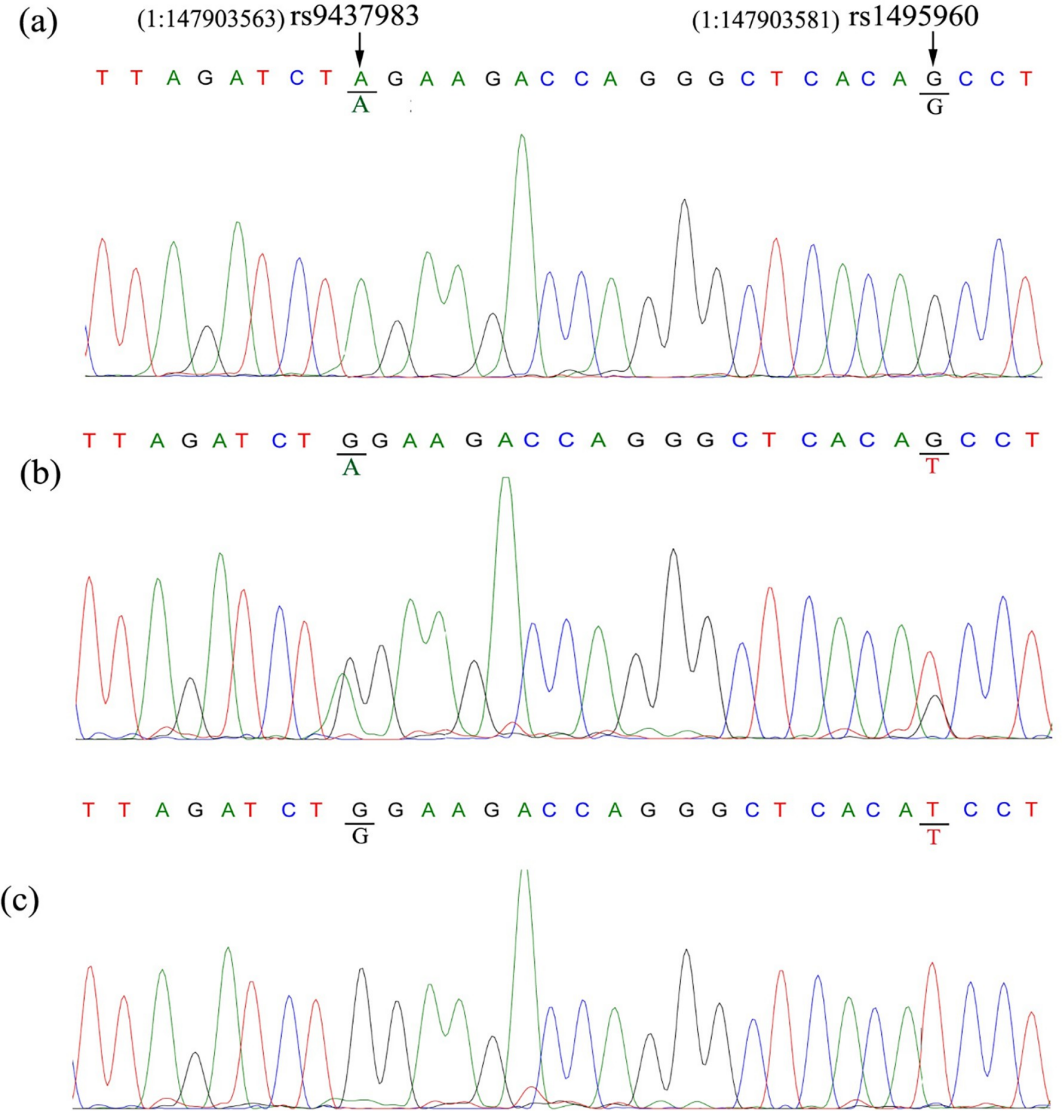
DNA sequencing results of SNP rs9437983 and rs1495960. (a) Chromatogram shows the Homozygous AA and GG genotypes of SNP rs9437982 and rs1495960 respectively. (b) Chromatogram shows the Heterozygous G/A and G/T genotypes of SNP rs9437983 and rs1495960 respectively. (c) Chromatogram shows the Homozygous GG and TT genotypes of SNP rs9437983 and rs1495960 respectively.

This research study was conducted to predict the age related cataract by using *GJA8* SNPs in. 400 patients suffering from age-related cataracts and 318 healthy individuals with no lens opacity were selected for this study. Two SNPs of *GJA8* A/G (rs9427983) and T/G (rs1495960) were studied. Figs 2 and 3 represent the results of TETRA-ARMS PCR products resolved on 2% agarose gels and Fig 4 represents the results of DNA sequencing of SNPs rs9437983 and rs1495960.

Table 3 indicates that both SNPs are in Hardy Weinberg equilibrium. Table 4 indicates the statistical description of the different variables used in this study. The results suggest that the gender representation was comparable among males and females. Further, it is also quite visible that the chances of cataracts are higher with the increase in age (*p-value < 0*.*00001*), as 66.4% and 93.9% of individuals in age groups 40–60 and 60–70, respectively, exhibited disease phenotype. Similarly, all individuals with a very high BMI (26 to 40) had the phenotype. Moreover, the chances of cataracts seemed to be relatively higher in individuals with diabetes, i.e., 90.7% (*p-value < 0*.*00001*). A high percentage of people suffering from cataracts also suffer from blood pressure abnormalities (*p-value < 0*.*00001*).

**Table 3:** Distribution of genotypic and allelic frequencies in cases and controls.

SNP	Genotype Allele	Controls (n = 318)	Cases (n = 400)
*GJA8* A/G (rs9437983)	AA A/G GG	180 (56.60%) 122 (38.3%) 16 (5.86%)	64 (16%) ^a^ 196 (49%) 140 (35%)
	Chi-Square value	0.653	0.112
	P-value	0.721 2df^b^	0.945 2df^b^
	A G	482 (75.79%) 154 (24.21%)	324 (40.5%) 476 (59.5%)
*GJA8* T/G (rs1495960)	TT T/G GG	73 (22.9%) 162 (50.9%) 83 (26.1%)	68 (17%) 197 (49.25%) 135 (33.7%)
	Chi-Square value	0.125	0.072
	P-value	0.939 2df^b^	0.964 2df^b^
	T G	308 (48.4%) 328 (51.5%)	333 (41.6%) 467 (58.3%)

a Parenthesis shows frequencies of individuals

b Degree of Freedom, *** P < 0.05.

**Table 4:** Descriptive analysis (%) of demographic variables.

Variables	Categories	Phenotype		p-value^+^
		No	Yes	
gender	Female*	146(39.7%)	222(60.3%)	0.011
	Male	172(49.1%)	178(50.9%)	
Age	20–30*	2(20%)	8(80%)	0.000
	20–40	232(72%)	90(28%)	
	40–60	76(33.6%)	150(66.4%)	
	60–70	8(6.1%)	124(93.9%)	
	70+	0(0%)	28(100%)	
BMI	06 to 15*	226(43%)	300(57%)	0.004
	16 to 25	92(50.5%)	90(49.5%)	
	26 to 42	0(0%)	10(100%)	
H/O Diabetes	No*	310(49.1%)	322(50.9%)	0.000
	Yes	8(9.3%)	78(90.7%)	
H/O Blood Pressure	No*	304(49.7%)	308(50.3%)	0.000
	Yes	14(13.2%)	92(86.8%)	
Use of Vitamins	No*	312(44.6%)	388(55.4%)	0.343
	Yes	6(33.3%)	12(66.7%)	
Tranquilizers	No*	318(46.1%)	372(53.9%)	0.000
	Yes	0(0%)	28(100%)	
Smoking	No*	304(48.7%)	320(51.3%)	0.000
	Yes	14(14.9%)	80(85.1%)	
Radiation Exposure	No*	314(46.3%)	364(53.7%)	-0.000
	Yes	4(10%)	36(90%)	
Steroids Medications	No*	318(46.8%)	362(53.2%)	0.000
	Yes	0(0%)	38(100%)	
GJA8 rs9437983	AA	180(73.8%)	64(26.2%)	0.000
	AG*	122(38.4%)	196(61.6%)	
	GG	16(10.3%)	140(89.7%)	
GJA8 rs1495960	TT	73(51.8%)	68(48.2%)	0.035
	TG	162(45.1%)	197(54.9%)	
	GG*	83(38.1%)	135(61.9%)	
Haplotypes	AAGG*	52(65.8%)	27(34.2%)	0.000
	AATG	74(69.2%)	33(30.8%)	
	AATT	54(93.1%)	4(6.9%)	
	AGGG	27(28.7%)	67(71.3%)	
	AGTG	77(46.1%)	90(53.9%)	
	AGTT	18(31.6%)	39(68.4%)	
	GGGG	5(11.1%)	40(88.9%)	
	GGTG	10(11.8%)	75(88.2%)	
	GGTT	1(3.8%)	25(96.2%)	

^+^All the values were calculated using chi-square test.

*The variables used as references in the regression models.

Additionally, a large proportion of individuals with a smoking habit were suffering from cataracts. These results suggest that the unhealthy living style could possibly increase the chances of getting a cataract. Most importantly, 100% relying on the steroid medication intake showed diseases phenotype.

The results also show that genotype (GG) of SNP GJA8 rs9437983 had the more number of reported cases than the alternative genotypes, suggesting that the GG genotype or in other words G allele might be risk allele to cataracts. Similar results were also observed in the individuals with the heterozygous genotype state (A/G) for this SNP, further strengthening the possibility of the G allele as the risk allele. Different regression and machine learning models estimated the effects of various demographic variables and genotypes. The GG genotype form of the SNP rs1495960 was more frequent in the cases than the alter genotype TT and G allele might be risk for the age-related cataract. Furthermore, the heterozygous seemed to be associated with the disease, as 54.9% of the individuals with this genotype had the disease phenotype.

The Allelic frequency distribution of *GJA8* SNPs rs9437983 and rs1495960 in different populations from 1000 genome is compared in Table 5.

**Table 5:** Allelic frequency distribution of *GJA8* SNPs rs9427983 and rs1495960 in different populations.

Population/ Country	*GJA8* A/G (rs9437983)		*GJA8* T/G (rs1495960)		Reference
	A%	G%	T%	G%	
Southern Punjab/Pakistan	74%	26%	48	52%	Present Study
African	76%	24%	74%	26%	1000 Genomes Project Phase 3
African Ancestry in Southwest	64%	36%	75%	25%	
African Caribbean in Barbados	74%	26%	75%	25%	
Luhya in Webeye, Kenya	72%	27%	77%	23%	
American	61%	39%	51%	49%	
Mexican Ancestry in Los Angelas	57%	43%	53%	47%	
Puerto Rican in Puerto	62%	38%	51%	49%	
Peruvian in Lima	59%	41%	51%	49%	
East Asian	59%	41%	54%	46%	
Han Chinese in Beijing	55%	45%	57%	43%	
Japanese in Tokyo, Japan	67%	33%	51%	49%	
European	71%	29%	48%	52%	
Northern and Western European Ancestry	62%	38%	53%	47%	
British in England and Scotland	69%	31%	55%	45%	
South Asian	82%	18%	49%	51%	
Bengali in Bangladesh	77%	23%	49%	51%	
Indian Telegu in the UK	84%	16%	44%	56%	
Sri Lankan Tamal in UK	82%	18%	49%	51%	

Table 6 outlines the results of the model fitting through the LR of two models (mentioned in the methodology). LR tests the probability of the presence or absence of an event, like in this case, the presence or absence of a cataract was evaluated. In both models, the categories used as references are marked with * in Table 4. The results indicated that variables like gender, diabetes status, blood pressure, radiation, and smoking significantly affect cataract phenotype. Both models, i.e., 1 and 2, seemed to be consistent in this regard as the explanatory demographic variables were the same in both. Furthermore, model 1 also indicates that the effect of the SNPs rs9437983 and rs1495960, including their genotypes, were also significant. On the other hand, model 2 shows that all haplotypes, except AATG, have a significant role in the model.

**Table 6:** Logistic regression model.

Variables	Category	Model 1			Model 2		
		Coef.	OR [95% CI of OR]	p-value	Coef.	OR [95% CI of OR]	p-value
(Intercept)		-1.270	0.28 [0.47–0.17]	0.000	-1.304	0.27 [0.51–0.14]	0.000
Gender	Male	-0.788	0.45 [0.7–0.29]	0.000	-0.764	0.47 [0.72–0.3]	0.001
BMI	26–35	-0.019	0.98 [1.57–0.61]	0.936	-0.072	0.93 [1.5–0.58]	0.768
	36–45	18.542	-	0.991	20.012	-	0.990
H.O. Diabetes	Yes	1.853	6.38 [15.35–2.65]	0.000	1.819	6.16 [15.22–2.5]	0.000
H.O. Blood Pressure	Yes	1.726	5.62 [11.28–2.8]	0.000	1.784	5.95 [12.25–2.89]	0.000
Use of Vitamins	Yes	1.256	3.51 [10.87–1.13]	0.029	1.295	3.65 [11.65–1.14]	0.029
Use of Tranquilizers	Yes	18.266	-	0.984	18.034	-	0.984
Smoking	Yes	2.292	9.9 [20.8–4.71]	0.000	2.276	9.74 [20.76–4.56]	0.000
Radiation Exposure	Yes	2.448	11.56 [37.41–3.57]	0.000	2.333	10.31 [32.8–3.24]	0.000
Steroids Medications	Yes	18.224	-	0.982	18.925	-	0.981
GJA8 rs9437982	AA	1.550	4.71 [7.49–2.96]	0.000	-	-	-
	GG	3.541	34.49 [68.6–17.34]	0.000	-	-	-
GJA8 rs1495960	TG	-0.536	0.58 [0.93–0.37]	0.024	-	-	-
	TT	-0.664	0.51 [0.94–0.28]	0.031	-	-	-
Haplotypes	AATG	-	-	-	-0.034	0.97 [2.15–0.44]	0.933
	AATT	-	-	-	-2.202	0.11 [0.53–0.02]	0.006
	AGGG	-	-	-	1.719	5.58 [12.17–2.56]	0.000
	AGTG	-	-	-	0.848	2.34 [4.66–1.17]	0.016
	AGTT	-	-	-	1.372	3.94 [9.62–1.61]	0.003
	GGGG	-	-	-	2.979	19.67 [64.46–6.01]	0.000
	GGTG	-	-	-	3.076	21.67 [54.98–8.54]	0.000
	GGTT	-	-	-	3.959	52.4 [435.88–6.3]	0.000

**Fig 5:**
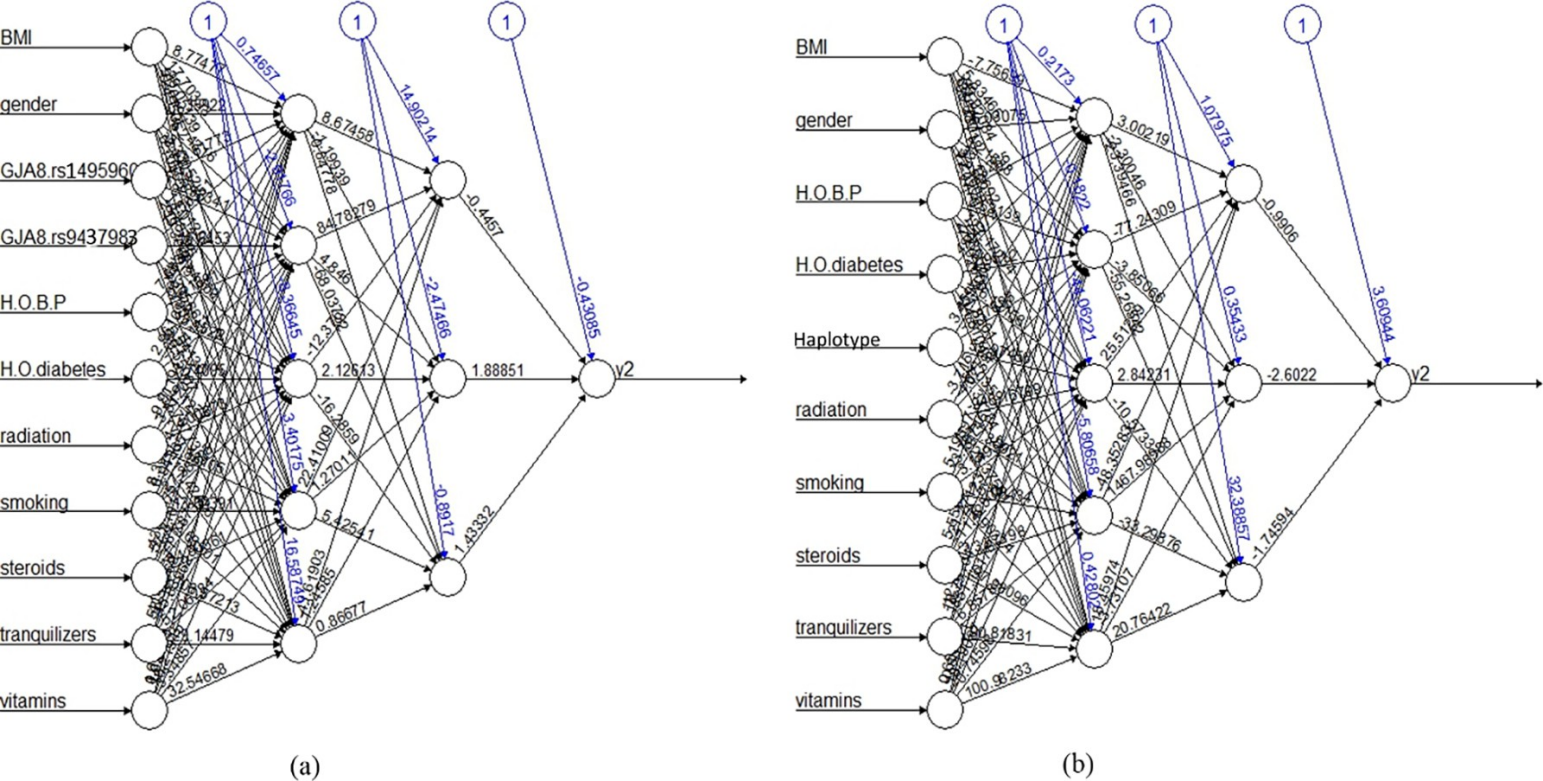
ANN plot with final weights. (a) plot for model 1 (b) plot for model 2.

ANN machine learning model is an important aspect of this study. As indicated in Fig 5, ANN models work by establishing different layers, including input, hidden, and output layers. The estimated final wights of each layer are also shown in these figures.

RF models are also quite effective in estimating the importance of a variable in a model. This importance is generally assessed in terms of the Gini coefficient, where a mean decrease in the coefficient indicates the importance of a particular variable in the model. The higher the value of mean decrease accuracy or mean decrease Gini score, the higher the importance of the variable in the model.

**Fig 6:**
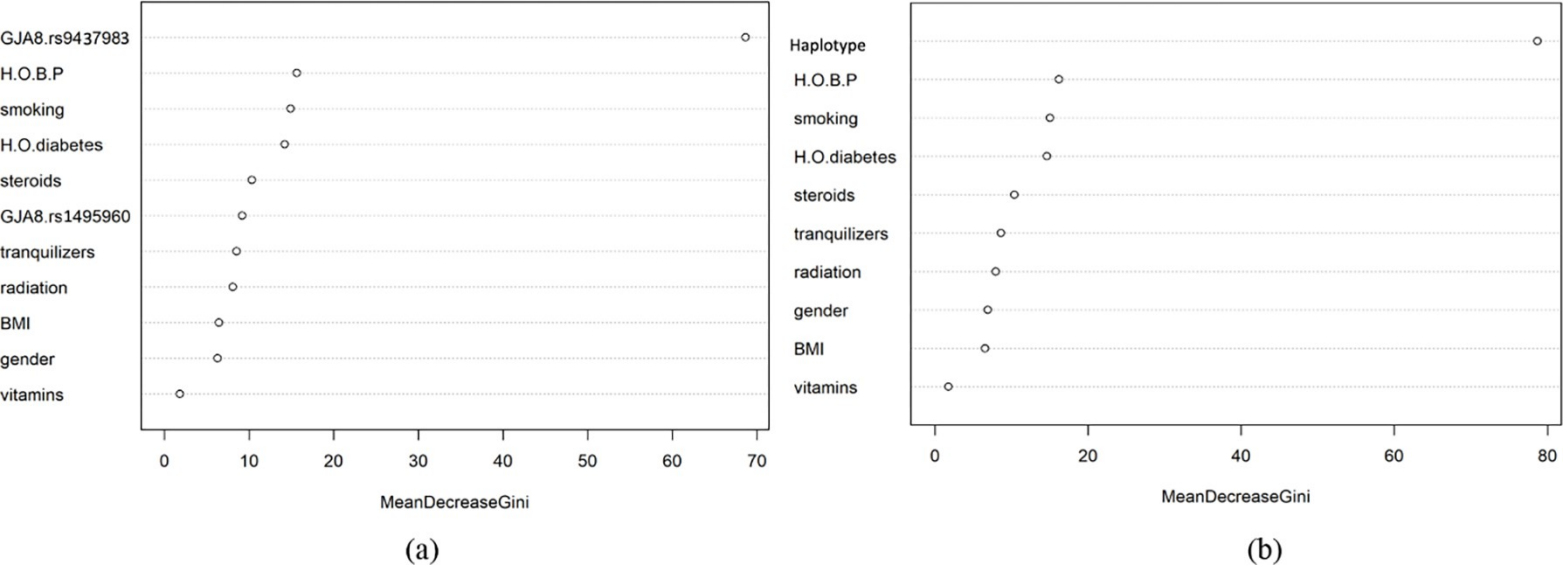
Importance and contribution of variables in Random Forest (RF) model.

Fig 6(A) indicates the mean decrease in the Gini coefficient is the highest for SNP rs9437983, suggesting an association between cataracts and the GJA8 gene. Fig 6(B) shows the importance of haplotypes in predicting cataracts. Age is also the second most crucial factor, even in the case of model 2. The results of both models show that the *GJA8* gene is associated with cataracts.

The main idea behind these models is to be able to classify the presence or absence of cataracts. Hence, it is vital to assess the performance of all three approaches in cataract predictions. In this regard, the AUC score and ROC curves were evaluated. Table 7 indicates AUC values for all models with three approaches.

**Table 7:** Comparison of machine learning models.

Models	AUC	AUC with 10-Fold CV	95% CI of AUC	Sensitivity	Specificity
LRM1	0.865	0.888	0.811–0.918	0.814	0.819
LRM2	0.870	0.898	0.819–0.921	0.824	0.805
ANN1	0.666	0.653	0.587–0.746	0.120	1.000
ANN2	0.503	0.550	0.414–0.592	0.916	0.111
RF1	0.819	0.832	0.763–0.875	0.777	0.861
RF2	0.819	0.843	0.762–0.876	0.805	0.833

A higher AUC value indicates that a model is better at distinguishing between two classes, i.e., the presence or absence of cataracts. The results in Table 7 suggest that the AUC values for models based on LR are the highest, followed by RF and ANN at the end. Moreover, the AUC values of RF models are more elevated than ANN-based models, indicating that RF models are better than ANN models. Sensitivity and specificity values indicate the model’s power to distinguish between the presence or absence of a cataract. The sensitivity indicates the model’s ability to associate a diseased person in a diseased category, and specificity suggests the model’s ability to associate a non-diseased person with a non-diseased category.

**Fig 7:**
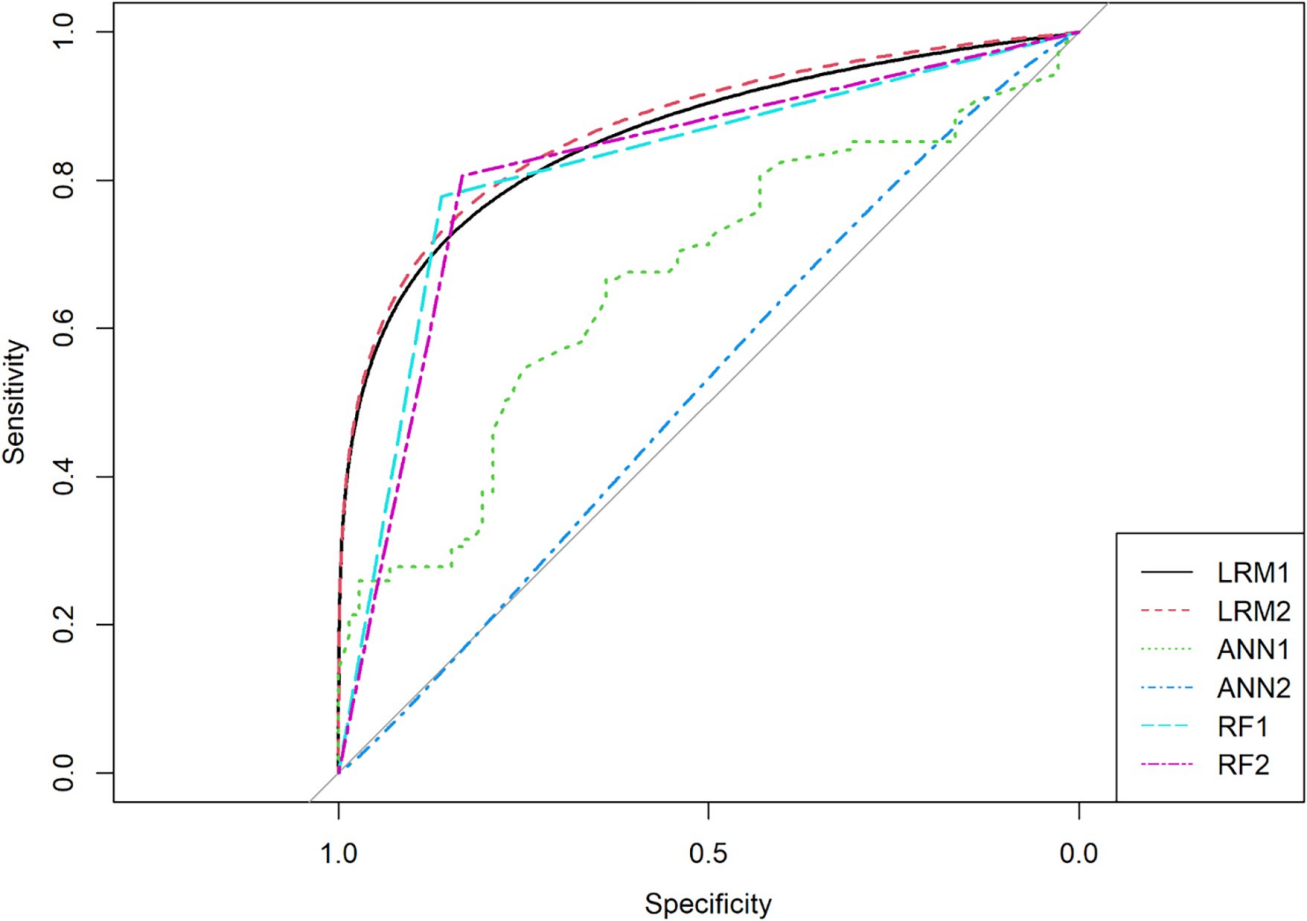
ROC curves.

Fig 7 also corroborates the results of Table 7. The ROC curves for both models based on the logistic regression are almost the same. Moreover, the RF models are closer to LR models. Both models of the RF approach give similar ROC curves. The curves seem to be strange for both ANN models.

## Discussion

Cataracts are the second most common eye impairments worldwide. They are a cause of both reversible and irreversible blindness. Cataracts can be regarded as multi-factorial disorders as several demographic and genetic factors are responsible for them. Cataracts are generally divided into several categories depending on their origin. One of the most common types of cataracts is age-related cataracts. Age-related cataracts are more prevalent in older ages [33]. The previous studies indicated that eight genes are associated with the age-related cataract including *EPHA2*, *GJA8*, *GALT*, *SLC16A12*, *HSF4*, *GALK1*, *FTL*, and *CRYAA* [38]. *GJA8* gene is of particular interest for this study as it produces gap junction proteins responsible for lens transparency. In this study, we predicted the age-related cataract by using association of different SNPs and Haplotypes of the *GJA8* gene with the age-related cataract using machine learning methods.

This study includes 718 individuals with 400 age-related cataract patients and 318 healthy individuals as control. The individual data comprised 146 and 172 healthy females and males, respectively, while 222 and 178 diseased females and males, respectively. In terms of age, the individuals ranged from 20 years to more than 70 years. To accurately predict the possibility of age-related cataracts, data about 10 demographic factors were also collected from all of the individuals. Moreover, the genetic data was obtained at SNP and haplotype levels by extracting DNA from the blood of the individuals.

Several demographic factors were found to be significantly associated with the disease. The results showed that gender is also associated with the age-related cataract, with females suffering from age-related cataracts more than males. Age appeared to be the most contributing factor to age-related cataracts as well. More than 66% of the individuals in the 40 to 60 years age group were suffering from age-related cataracts, and this prevalence reached 93.9% in the 60 to 70 years age group. The prevalence of age-related cataracts in old age groups has already been higher than in younger age groups [33], indicating a high impact of age. Other health-related factors also appeared to be quite associated with age-related cataracts. For example, more than 90% of the patients with age-related cataracts also had diabetes. These results are in line with some previous studies where the prevalence of age-related cataracts was found to be higher in diabetic patients [9,39]. Age-related cataract patients also suffered from blood pressure issues, as 90.7% of age-related cataract patients also had blood pressure issues. It has also been reported that hypertension and blood pressure can impact the conformation of eye lens proteins, resulting in cataracts [40]. Age-related cataract is not only caused by demographic factors but also by genetic factors. *GJA8* gene has been reported to be associated with age related cataract. Thus, both demographic and genetic factors are important for this study.

The main idea of this study was to predict age-related cataracts. The data was divided into two categories, demographic factors, and SNPs (model 1) and the other was demographic factors and haplotypes (model 2). Three different statistical and supervised machine learning approaches were tested in this study to predict age-related cataracts. The first approach was based on LRM, the second one was RF, and the third was ANN models. We tested SNPs (rs9437983 and rs1495960) and haplotypes of the *GJA8* gene along with demographic factors to develop prediction models. These statistical and machine learning approaches have been implied for age-related cataract risk factors detection [41], diagnosis [42], and post-surgery risks [7]. This research study is unique in a way that it tries to predict the possibility of age-related cataract development based on several demographic and genetic factors.

All models were compared for their prediction power using AUROC analysis. The AUC indicates that both LRM1 and LRM2 had the highest AUC value of 0.865and 0.870 respectively, along with specificity and sensitivity values of more than 0.80. Based on AUC value, RF models (RF1 and RF2) were the second-best models after LRMs. ANN-based models (ANN1 and ANN2) were the least effective in this case for cataract prediction because their AUC, sensitivity, and specificity values were relatively lower than LRMs and RFs. LRMs have already been found to be quite efficient as they successfully capture the effects of demographic and genetic factors. LRMs are parametric models, which might be the reason for their better predictive abilities compared to nonparametric models like RF and ANN. However, an advantage of RF is its ability to attach differential weights to different predictors depending on their importance. Moreover, ANN models tend to underperform in many cases as they are extremely difficult to parametrize because they need a large amount of data beforehand in the learning stage [43]. The LR-based models developed in this study were efficient in correctly predicting age-related cataracts. Applying such machine learning tools can be pretty effective in timely predicting the possibility of a cataract and proposing measures to control cataract formation.

All three machine learning approaches indicated that both demographic and genetic factors are associated with age-related cataracts. A significant association between gender and age-related cataract was observed in both models. The female gender was more prone to age-related cataracts than the male gender. Similar effects of gender were also observed in a study on a Korean population [44]. Similarly, age, diabetes disease status, blood pressure, and radiation interception turned out to be significantly associated with age-related cataracts in both models.

The homozygous genotype GG of rs9437983 appeared to be a risk allele as 89.7% of individuals with this genotype were suffering from cataracts, but the genotype AA of the same SNP appeared to be quite resistant to cataracts with only 26.2% of disease cases. In case of SNP rs1495960 genotypic frequency was different among patients and controls. Genotype TT was more frequent in controls (51.9%) while in patients it was 48.1% genotype. GG of rs1495960 appeared to be a risk allele as 61.9% of individuals with this genotype were suffering from cataracts as frequency of GG genotype was 38.1% in controls. Heterozygous genotype T/G was more frequent in patients (68.9%) while in controls it was 45.1%. These results recommend that cataract patients were carriers of mutant G allele and G allele was a risk allele. The results of SNP A/G (rs9437983) and SNP T/G (rs1495960) were different from Chinese population. They found no difference of genotype frequencies among patients and controls [21].

Haplotypes AGGG, AGTG, AGTT, GGGG, GGTT, and GGTG are more highly distributed in cases than in controls and these haplotypes increased the risk of disease. Individuals with haplotypes GGTT and GGTG, AGGG, GGGG are homozygous for risk G allele. This supports that the G allele is a risk allele for cataracts. While individuals with haplotypes AGTT, GGTG also carrier of mutant allele supports that G allele is a risk allele. Haplotypes AATT and AATG have higher distribution in controls than cases. Individuals with AATT and AATG haplotypes are homozygous for the ancestral A allele and have a low risk for cataracts.

In conclusion, we found that two SNPs T/G (rs1495960 and A/G (rs9437983) of the *GJA8* gene, previously reported in the Chinese population only, have a significant association with age-related cataracts in the population of southern Punjab Pakistan. The results were further confirmed by different supervised machine learning algorithms. By inclusion of demographic variables, it was also made possible to predict the possibility of age-related cataracts, which is a useful tool, particularly LR models, for medical staff to develop effective disease management techniques.

## Conclusion

Age-related cataract is considered the leading cause of senile blindness all over the world. The results indicated that the *GJA8* gene is significantly associated with the disease, with allele G of both SNPs (rs9437983 and rs1495960) and being risk allele for age-related cataracts in the Pakistani population Further, two models were developed to predict the age-related cataract by using demographic factors and genetic factors with the age-related cataract. In the first model, demographic factors and the SNPs of the *GJA8* gene were used to predict the age-related cataract, while in the second model the demographic factors and the haplotypes of the *GJA8* gene were used for prediction age-related cataract. Both models were fitted using three supervised machine learning methods including LRM, ANN, and RF. Both LRMs, based on SNPs and Haplotypes, turned out to be the best in cataract prediction as their AUC values were the highest, i.e., 0.865 and 0.870. The LRM model based on demographic factors and haplotypes was considered as a best selected model for the prediction of age-related cataract The limitation of our study is that we enrolled some individuals in control group that were younger than the individuals in cases. Although, these control group individuals did not have series of demographic and clinical factors associated with age related cataract, but it might be possible that some uncertainty may occur. Due to limitation of time for this study we were unable to find the normal individuals of same age group. So, this factor can be considered in future studies.

## Supporting information

S1 Raw images (PDF)
