# Supplementary figures and images for: Potential of GJA8 gene variants in predicting age-related cataract: A comparison of supervised machine learning methods
Source: PLoS One. 2023 Aug 31;18(8):e0286243. doi: 10.1371/journal.pone.0286243 (PMC10470928; doi:10.1371/journal.pone.0286243)

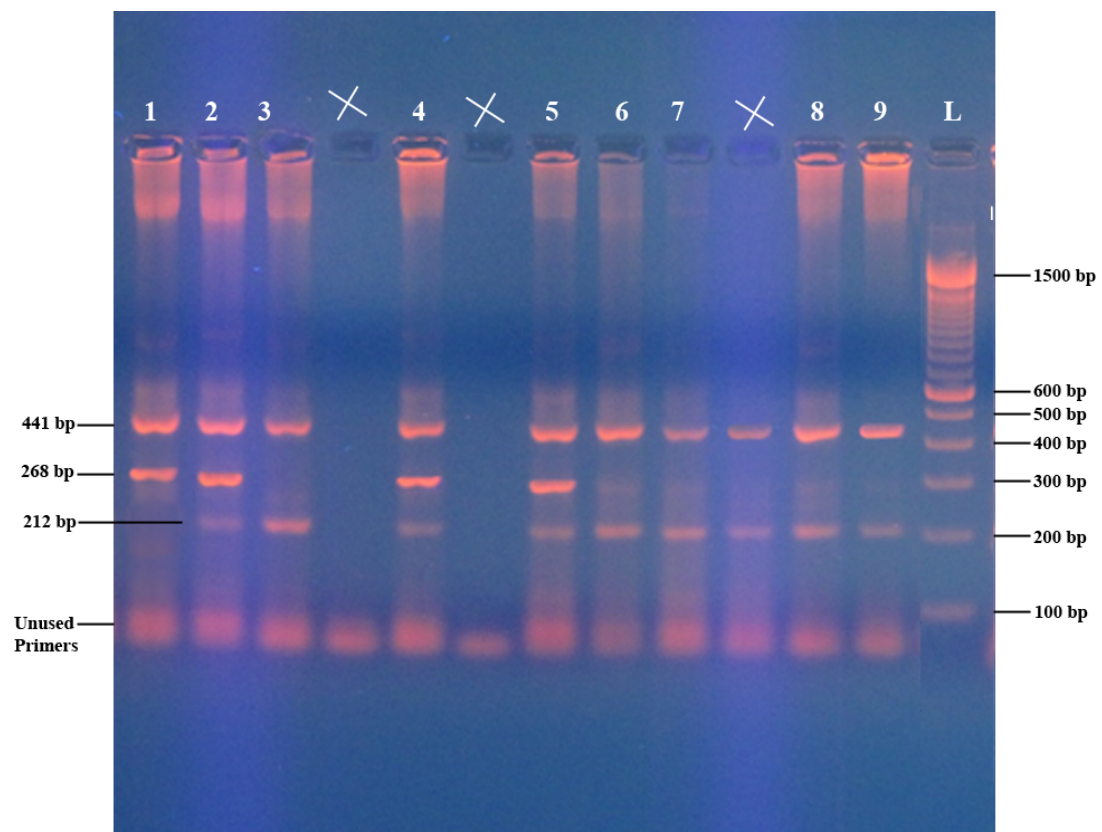

**FIG 2**

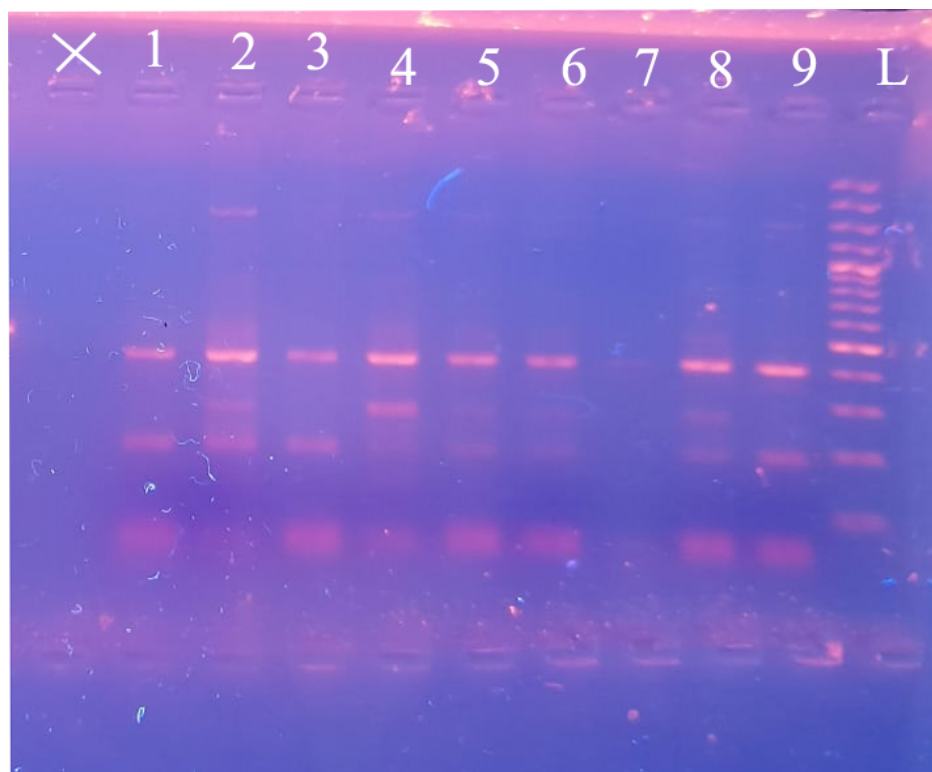

**Fig. 3**

Supplement: S1 Raw images — (PDF) [file pone.0286243.s001.pdf]
